# A Segment-Based Trajectory Similarity Measure in the Urban Transportation Systems

**DOI:** 10.3390/s17030524

**Published:** 2017-03-06

**Authors:** Yingchi Mao, Haishi Zhong, Xianjian Xiao, Xiaofang Li

**Affiliations:** 1 College of Computer and Information, Hohai University, Nanjing 210098, China; yingchimao@hhu.edu.cn (Y.M.); zhonghs@hhu.edu.cn (H.Z.); 2 School of Computer Information & Engineering, Changzhou Institute of Technology, Changzhou 213032, China; xiaoxj@czu.cn

**Keywords:** GPS trajectory, GPS sensor, trajectory similarity measure, spatial-temporal data

## Abstract

With the rapid spread of built-in GPS handheld smart devices, the trajectory data from GPS sensors has grown explosively. Trajectory data has spatio-temporal characteristics and rich information. Using trajectory data processing techniques can mine the patterns of human activities and the moving patterns of vehicles in the intelligent transportation systems. A trajectory similarity measure is one of the most important issues in trajectory data mining (clustering, classification, frequent pattern mining, etc.). Unfortunately, the main similarity measure algorithms with the trajectory data have been found to be inaccurate, highly sensitive of sampling methods, and have low robustness for the noise data. To solve the above problems, three distances and their corresponding computation methods are proposed in this paper. The point-segment distance can decrease the sensitivity of the point sampling methods. The prediction distance optimizes the temporal distance with the features of trajectory data. The segment-segment distance introduces the trajectory shape factor into the similarity measurement to improve the accuracy. The three kinds of distance are integrated with the traditional dynamic time warping algorithm (DTW) algorithm to propose a new segment–based dynamic time warping algorithm (SDTW). The experimental results show that the SDTW algorithm can exhibit about 57%, 86%, and 31% better accuracy than the longest common subsequence algorithm (LCSS), and edit distance on real sequence algorithm (EDR) , and DTW, respectively, and that the sensitivity to the noise data is lower than that those algorithms.

## 1. Introduction

With the rapid development of sensors technology and the popularization of personal smart devices, GPS sensors are widely used to track moving objects, such as people, cars, and animals. A large number of trajectory data emerges every day. The trajectory data from GPS sensors are the spatio-temporal data sequences of mobile objects with the space-time variation. With the development of the Internet of Things, urban computing, and other research fields, the analysis of spatio-temporal data-based transportation systems have become a hot topic in the fields of machine learning. The trajectory data analysis can be a great driving force for all of the fields, for example, through applying the trajectory similarity measure algorithm, the distance matrix can be computed, which can be used to cluster the trajectory of peoples’ activities for finding the popular routes and hot spots and visualizing in OpenStreetMap [1,2]. In the intelligent transportation systems, it is of great practical value to measure the similarity of the trajectories of moving objects in a real-time, accurate, and reliable way. Intelligent trajectory measurement cannot only provide accurate location-based services, but also monitor and estimate traffic jams [3].

In trajectory data mining, one of the most important and fundamental works is to compute the similarity between different trajectories. Based on the similarity measurement of trajectory data, the trajectories can be clustered, classified, and retrieved [4]. The accuracy of the similarity measurement significantly affects the accuracy of the trajectory data mining. In recent years, some mainstream algorithms for trajectory similarity measurement have been proposed, such as the dynamic time warping algorithm (DTW) [5], longest common subsequence algorithm (LCSS) [6], and edit distance on real sequence algorithm (EDR) [7]. Those algorithms can obtain the results of similarity measurement through computing spatial point-to-point distances or temporal distances. However, there are common drawbacks resulting in the low accuracy. For example, the DTW algorithm just directly calculates the point-point distance, ignoring the influence of the different trajectory sampling methods on the generated trajectory sequence. The LCSS algorithm neglects optimizing the temporal distance of the trajectory data. The EDR algorithm does not consider the trajectory shape factor. In order to improve the accuracy, a segment-based dynamic time warping algorithm (SDTW) is proposed to measure the trajectory similarity. First, the proposed SDTW adopts the point-segment distance to reduce the sensitivity influence from the trajectory sampling methods. Then, considering the temporal distance factor, SDTW introduces the prediction distance to convert the temporal distance into the spatial distance. Finally, SDTW introduces the segment-segment distance to improve the computation accuracy by adjusting the parameters of shape factors.

The remainder of this paper is organized as follows. Section 2 discusses the related work and analyzes their drawbacks. Some definitions and problem statements are described in Section 3. Section 4 presents the proposed SDTW algorithm, and the performance evaluations are given in Section 5. Discussion and conclusions are given in Section 6.

## 2. Related Work

Trajectory sequence data can be regarded as time sequence data. Many approaches to the trajectory similarity measurement are introduced from the similarity measurement to the time sequence data. The simplest trajectory similarity measurement is the Euclidean distance, but it cannot obtain better accuracy when the local time shifts or when those trajectories lack the same length [8]. In order to improve the accuracy of similarity measurement, the dynamic time warping algorithm (DTW), longest common subsequence algorithm (LCSS), and edit distance on real sequence algorithm were proposed and widely applied.

Based on the idea of dynamic programming to find the optimal match point pairs between the trajectory points, the DTW can effectively solve the problem of local time shifting and various trajectory lengths [5]. The DTW algorithm was firstly introduced for speech recognition, then applied to the time sequence analysis later. The LCSS adopted a threshold ε to identify the match point pairs [6], but it is a similarity measurement in rough granularity without considering non-match pairs of points. The EDR is an edit distance-based algorithm, which uses a threshold ε to identify the match point pairs and the non-match points, different from the LCSS. Those similarity measurement algorithms can be divided into two types [8]: the one based on *L*1 and *L*2 paradigms, such as the DTW; and the other computing similarity scores based on the matching threshold, such as the LCSS and the EDR.

Wang et al. have evaluated the performance on the accuracy of main similarity measurement algorithms, DTW, LCSS, ERP [9], EDR, and SpaDe [10], in the different time sequence datasets [11]. The experimental results demonstrate that the DTW algorithm can obtain the most accurate results of the similarity measurement in the majority of datasets although its computation speed is slow. Based on the evaluation results, many similarity measurement algorithms, such as Kim [12], Keogh [13], and Improved [14], have been proposed to reduce the computation complexity at the same measurement accuracy as the DTW algorithm.

From the above analysis, it can be found that those algorithms have common drawbacks affecting the accuracy of similarity measurement.

**Figure 1:**
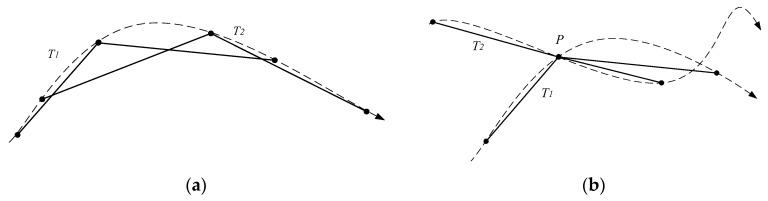
Various point sampling methods of the same trajectory. (**a**) A trajectory of two sampling methods; and (**b**) two trajectories take the same point.

(1) The DTW, LCSS, and EDR algorithms only consider the comparison of two individual points. In fact, different sampling methods can form different trajectory sequences, which results in a significant negative impact on the final measurement results [15]. As shown in Figure 1a, the trajectory sequence data of a curve trajectory with an arrow may have two-point sampling methods *T*_1_ and *T*_2_. Two original trajectories are essentially identical, but their trajectory sequences are quite different. In Figure 1b, two trajectories intersect at point *P*, and their trajectory sequences $T_{1}$ and $T_{2}$ sample the point *P*. An obvious difference between the two trajectories is produced, but the difference is weakened due to the intersection point *P*. Thus, computing the trajectory similarity completely based on the discrete trajectory points will cause the loss of the details of the trajectories. It is necessary to find a way to keep the details to a certain extent.
(2) Only considering the distances between the pairs of points, the mentioned algorithms cannot take shape factors into account [16]. However, shape factor is an important feature of a natural trajectory. It may result in the loss of computation accuracy when the shape factors are ignored.
(3) Most algorithms of similarity measurement are derived from the time sequence similarity computation without considering the temporal distance computation between two trajectory points. Since the time measurement is different from the space measurement, it makes no sense just to simply add two weights. To solve that problem, Lee et al. proposed a trajectory distance measurement method with the weighted addition of the parallel distance, the perpendicular distance, and the angle distance [1]. Unfortunately, the proposed measurement method by Lee et al. cannot solve problems (1) and (2).

To solve the above three problems, a segment-based trajectory similarity measurement algorithm is proposed to improve the accuracy.

## 3. Problems and Definitions

Mobile objects generally have time and space attributes, respectively. Space attributes can be three-dimensional or two-dimensional. Two-dimension is the most widely used, so all of the words “space” refers to two-dimension space in this paper. A trajectory records a continuous movement trace of a mobile object. Due to the limitations of the GPS sensors, a trajectory *T* consists of a series of points (x,y,t), where (x,y) is the spatial recorded point, *t* is the recorded time. For convenience, a natural trajectory and a trajectory sequence are strictly distinct.

**Definition 1** (**natural trajectory**)**.** *A continuous trajectory of a mobile object*.

**Definition 2** (**trajectory sequence**)**.** *With a given Euclidean space, a natural trajectory can be expressed as $T=\{P_{1},P_{2},...,P_{n}\}$, where the discrete trajectory points are ordered by time, $P_{i}$ refers to the trajectory point i, $P_{i}=(x_{i},y_{i},z_{i})$, and n represents the number of points in the trajectory. T is the recorded trajectory sequence from the natural trajectory*.

**Definition 3** (**sub-trajectory segment**)**.** *Two adjacent discrete trajectory points $P_{i}$ and $P_{i+1}$ are connected to form a trajectory segment $P_{i}P_{i+1}$, which is a sub-trajectory segment*.

**Definition 4** (**natural sub-trajectory segment**)**.** *A part of the natural trajectory between two adjacent discrete trajectory points is constructed as a natural sub-trajectory segment*.

**Figure 2:**
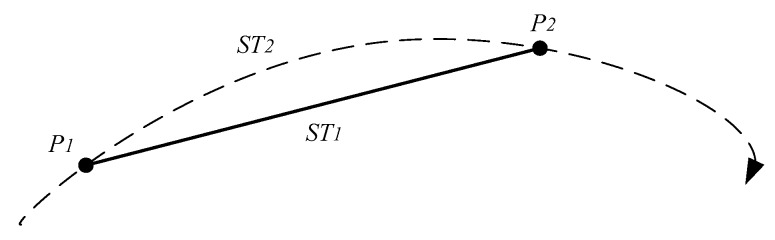
A sub-trajectory segment and a natural sub-trajectory segment.

A trajectory sequence consists of a series of discrete points. Two adjacent discrete points are connected to form a sub-trajectory segment. Moreover, a real trajectory segment must exist between two adjacent discrete points. In Figure 2, $ST_{1}$ and $ST_{2}$ are a sub-trajectory segment and a natural sub-trajectory segment, respectively.

**Figure 3:**
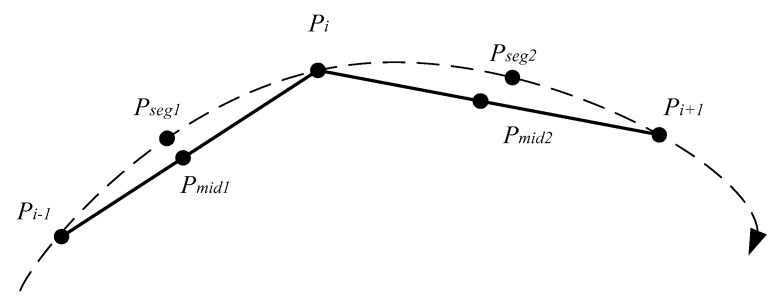
A Proxy of a natural sub-trajectory.

**Definition 5** (**proxy natural sub-trajectory segment**)**.** *In Figure 3, $P_{seg1}$ is denoted as a medium point of the natural sub-trajectory segment between $P_{i}$ and $P_{i-1}$. $P_{seg2}$ is denoted as the medium point of the natural sub-trajectory segment between $P_{i}$ and $P_{i+1}$. The proxy natural sub-trajectory segment of trajectory point $P_{i}$ is the natural sub-trajectory segment between $P_{seg1}$ and $P_{seg2}$*.

**Definition 6** (**proxy sub-trajectory**)**.** $P_{mid1}$ *is marked as a midpoint of the sub-trajectory segment of* $P_{i}$ *and* $P_{i-1}$*, and* $P_{mid2}$ *is marked as the midpoint of the sub-trajectory segment of* $P_{i}$ *and* $P_{i+1}$*. The sub-trajectory formed by* $P_{mid1}$ $P_{i}$ *and* $P_{i}$ $P_{mid2}$ *is the proxy sub-trajectory of* $P_{i}$.

A discrete trajectory sequence represents a whole natural trajectory. A trajectory point on its sequence represents a part of the natural trajectory, called as the proxy natural sub-trajectory of the point. A natural trajectory can only be stored as a trajectory sequence; thus, the proxy natural sub-trajectory segment cannot be obtained. It can only obtain the proxy sub-trajectory of the trajectory points.

The problem to be solved in this paper is to compute the distance Dist(R,S) between two given trajectory sequences *R* and *S*, where $R=\{P_{1},P_{2},...,P_{n}\}$ and $S=\{SP_{1},SP_{2},...,SP_{m}\}$. The longer the distance, the less similarity Sim(R,S).

## 4. SDTW Algorithm

Due to ignoring the relationship between a trajectory sequence and a natural trajectory, the current trajectory similarity measurement algorithms are sensitive to the sampling methods. To reduce the sensitivity of the points sampling methods, a point-point distance can be converted to a distance from a point to a specific segment, which is defined as a point-segment distance. There is a fundamental difference between the temporal distance and the spatial distance of trajectory points. In this paper, the time difference and trajectory’ shape are integrated to convert a temporal distance into a spatial distance and the prediction distance is presented. The DTW algorithm only uses the point-point distance, without considering the trajectory’s important characteristic—shape—which results in the low accuracy of the DTW algorithm. If the shape factors are included, the accuracy of the similarity measurement can be improved. A trajectory sequence is regarded as multiple continuous trajectory segments, the shape lies in the difference of an angle between trajectory segments. An included angle can be considered into the similarity calculation, and its result is the segment-segment distance.

The above three distances are integrated with the traditional DTW algorithm to propose a new segment–based dynamic time warping algorithm (SDTW). SDTW adopts the point-segment distance, prediction distance, and segment-segment distance to compute the accumulative distance of two trajectory sequences, which can improve the accuracy.

### 4.1. Point-Segment Distance

**Figure 4:**
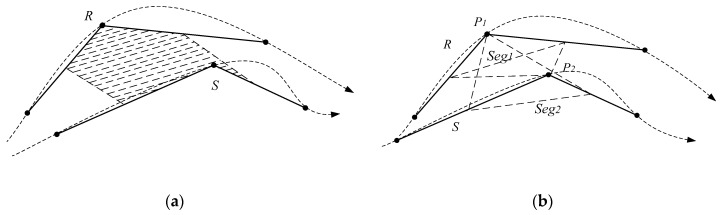
Two computation methods of the proxy sub-trajectory distance. (**a**) The area enclosed by the two proxy trajectories; and (**b**) a simplified calculation method.

The spatial distance of two trajectory points can be converted to the spatial distance of their proxy trajectories. In fact, the point-segment distance is the spatial distance of the pair of two trajectory points. The distance of the two proxy trajectories, $S_{true}$ is the area enclosed by them (Figure 4a). The plane is an irregular polygon area, the computation is difficult. The sum of $S_{1}$ enclosed by $P_{1}$ and $S{\mathrm{eg}}_{2}$ and $S_{2}$ enclosed by $P_{2}$ and $S{\mathrm{eg}}_{1}$ (Figure 4b) shows a positive correlation with $S_{true}$. That is, when the relative displacement of the two proxy trajectories occurs, the trend of $S_{1}$ + $S_{2}$ is the same as that of $S_{true}$. So, $S_{true}$ can be replaced with the sum of $S_{1}$ and $S_{2}$.

It is obvious that the distance calculation method based on the area is not an effective approach, especially for a trajectory point with a long proxy sub-trajectory, which results in a larger sum enclosed by it and other proxy trajectories. From the above analysis, the length of $Seg_{1}$ and $Seg_{2}$ shows a positive correlation with the condition of a trajectory point with a long proxy sub-trajectory. The longer a trajectory is, the worse the result is. It can adopt S/Seg to convert the spatial distance between $P_{1}$ and $P_{2}$ into the sum of the distance from $P_{1}$ and $S{\mathrm{eg}}_{2}$, and the distance from $P_{2}$ and $S{\mathrm{eg}}_{1}$. That is, S/Seg is the sum of point-segment distances.

**Figure 5:**
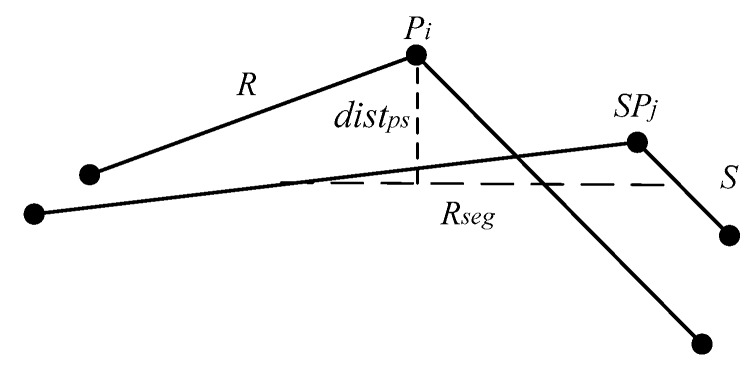
Point-segment distance.

Assume that $P_{i}\left(x_{i},y_{i}\right)$ is trajectory point *i* on the trajectory sequence *R*, and $SP_{j}\left(x_{j},y_{j}\right)$ is trajectory point *j* on the trajectory sequence *S*. Define $dist_{ps}\left(P_{i},SP_{j}\right)$ as the point-segment distance of $P_{i}$ and $SP_{j}$. Define $dist_{ps}\left(SP_{j},P_{i}\right)$ as the point-segment distance of $SP_{j}$ and $P_{i}$, and $dist_{ps}\left(P_{i},SP_{j}\right)\neq dist_{ps}\left(SP_{j},P_{i}\right)$. Figure 5 illustrates the point-segment distance computation.

To compute $dist_{ps}\left(P_{i},SP_{j}\right)$, it is first to compute the midpoint $P_{mid1}\left(x_{mid1},y_{mid1}\right)$ of $SP_{j}$ and $SP_{j-1}$, and the midpoint $P_{mid2}\left(x_{mid2},y_{mid2}\right)$ of $SP_{j}$ and $SP_{j+1}$. $P_{mid1}\left(x_{mid1},y_{mid1}\right)$ and $P_{mid2}\left(x_{mid2},y_{mid2}\right)$ can computed as follows Equation (1):

(1) $$\{\begin{array}{c} \left(x_{mid1},y_{mid1}\right)=\left(\left(x_{j-1}+x_{j}\right)/2,\left(y_{j-1}+y_{j}\right)/2\right)\\ \left(x_{mid2},y_{mid2}\right)=\left(\left(x_{j}+x_{j+1}\right)/2,\left(y_{j}+y_{j+1}\right)/2\right) \end{array}$$

Then it computes the shortest distance between $P_{i}$ and $R_{seg}$. $dist_{ps}\left(P_{i},R_{seg}\right)$ is [17]:

(2) $$dist_{ps}(P_{i},R_{seg})=\{\begin{array}{c} \sqrt{{\left(x_{i}-x_{mid1}\right)}^{2}+{\left(y_{i}-y_{mid1}\right)}^{2}}\;\;\;\;\;\;\;if\;\;r\leq 0\\ \sqrt{{\left(x_{i}-x_{mid2}\right)}^{2}+{\left(y_{i}-y_{mid2}\right)}^{2}}\;\;\;\;\;\;\;\;\;if\;\;\;r\geq L_{seg}\\ \;\;\;\;\sqrt{{\left(x_{i}-dx\right)}^{2}+{\left(y_{mid1}-dy\right)}^{2}}\;\;\;\;\;\;\;\;\;otherwice \end{array}$$

where $r=\left(x_{mid2}-x_{mid1}\right)\times \left(x_{i}-x_{mid1}\right)+\left(y_{mid2}-y_{mid1}\right)\times \left(y_{i}-y_{mid1}\right)$; $L_{seg}$ is the length of the derivation segment, $dx=(x_{mid1}+\left(x_{mid2}-x_{mid1}\right)\times \left(r/L_{seg}\right))$:$dy=(y_{mid1}+\left(y_{mid2}-y_{mid1}\right)\times \left(r/L_{seg}\right))$.

The formula for $dist_{ps}\left(SP_{j},P_{i}\right)$ is the same as $dist_{ps}\left(P_{i},SP_{j}\right)$, and the spatial distance $dist_{p}\left(P_{i},SP_{j}\right)$ between $P_{i}$ and $SP_{j}$ with the SDTW is as shown in Equation (3):

(3) $$dist_{p}\left(P_{i},SP_{j}\right)=dist_{ps}\left(P_{i},SP_{j}\right)+dist_{ps}\left(SP_{j},P_{i}\right)$$

### 4.2. Prediction Distance

Most of the trajectory similarity measurement algorithms are introduced from the time sequence similarity algorithms without considering to optimize the trajectory data. However, the time series data measurement and space measurement of the trajectory are essentially different, so it is necessary to figure out a solution to calculate the temporal distance integrated with spatial distance.

**Figure 6:**
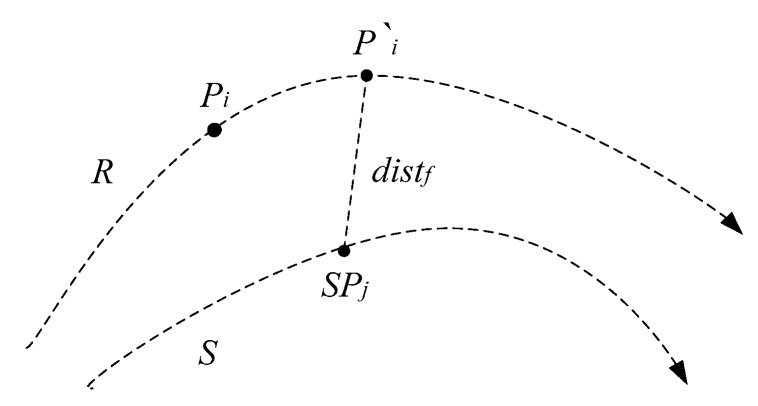
Prediction distance.

In Figure 6, the time distance between $P_{i}$ on trajectory *R* and $SP_{j}$ on trajectory *S* is computed. The timestamp of $P_{i}$ is $t_{i}$, the timestamp of $SP_{j}$ is $t_{j}$. The difference between $t_{i}$ and $t_{j}$ can actually be reflected on a specific trajectory. Assume that $P_{i}$ is regarded as a mobile object. When $t_{i}$ < $t_{j}$, its space location after the time interval $t_{j}-t_{i}$ is the space location of *R* at the timestamp $t_{j}$, known as a prediction position of $P_{i}$, denoted as $P_{i}\prime$.

The temporal distance between $P_{i}$ and $SP_{j}$ is converted into the spatial distance between the prediction location of $P_{i}\prime$ and $SP_{j}$ , known as the prediction distance. It can convert a temporal distance into a spatial distance, and reflect the time distance of trajectory points on the trajectory. It can be seen that he prediction distance has good interpretability. It can effectively improve the accuracy of similarity measurements. Therefore, the natural trajectory cannot be recorded, so the similarity measurement should be based on the trajectory sequence data.

Assume that $P_{i}\left(x_{i},y_{i},t_{i}\right)$ represents a trajectory point *i* on trajectory *R* and $SP_{j}\left(x_{j},y_{j},t_{j}\right)$ is a trajectory point *j* on trajectory *S*. To compute the prediction distance between $P_{i}\left(x_{i},y_{i},t_{i}\right)$ and $SP_{j}\left(x_{j},y_{j},t_{j}\right)$, one first compares the timestamps of $P_{i}$ and $SP_{j}$. The point with an earlier timestamp is set as *A*, and the other with the later timestamp as *B.* Their time difference is $\Delta t=t_{A}-t_{B}$.

The next step is to compute the prediction location of point *B* with the later timestamp, named as B′. Since the information stored in the trajectory sequence is limited and the positions of the moving object cannot be obtained at any time, the prediction location B′ is only an approximate position of point *B*. Then, it traverses the timestamp for each trajectory point to search the track range of point *B* at the timestamp $t_{B}+\Delta t$. Suppose at the timestamp $t_{B}+\Delta t$, point *B* is located between point i−1 and *i*. The spatial coordinates $\left(x_{B^{\prime }},y_{B^{\prime }}\right)$ of the prediction position B′ can be calculated as follows:

(4) $$\{\begin{array}{c} x_{B^{\prime }}=x_{i-1}+v_{i}^{x}\times \left(t_{B}+\Delta t-t_{i-1}\right)\\ y_{B^{\prime }}=y_{i-1}+v_{i}^{y}\times \left(t_{B}+\Delta t-t_{i-1}\right) \end{array}$$

Suppose that it is a uniform linear motion between any two points on the trajectory, it can compute the velocity between two points as follows:

(5) $$\{\begin{array}{c} v_{i}^{x}=\left(x_{i}-x_{i-1}\right)/\Delta t_{i}\\ v_{i}^{y}=\left(y_{i}-y_{i-1}\right)/\Delta t_{i} \end{array}$$

If there does exist the corresponding recorded trajectory point *B* at the timestamp $t_{B}+\Delta t$, the B′ can be estimated as follows:

(6) $$\{\begin{array}{c} x_{B^{\prime }}=x_{B}+\left(x_{N}-x_{1}\right)/\left(t_{N}-t_{1}\right)\times \Delta t\\ y_{B^{\prime }}=y_{B}+\left(y_{N}-y_{1}\right)/\left(t_{N}-t_{1}\right)\times \Delta t \end{array}$$

where *N* is the total number of the points on the trajectory, on which point *B* is located.

The prediction distance between *A* and *B* is calculated as follows:

(7) $$dist_{t}\left(A,B\right)=dist\left(A,B^{\prime }\right)$$

where $dist\left(A,B^{\prime }\right)$ is the Euclidean distance between *A* and B′ in the coordination.

The prediction distance between *A* and *B* also presents the point-segment distance between point *A* and segment *BB’*.

### 4.3. Segment-Segment Distance

Suppose that $S_{i}$ is one segment *i* on the trajectory *R*, and $SS_{j}$ is one segment *j* on the trajectory *S*. Suppose that $S_{i}$’s two endpoints are $P_{i}\left(x_{i},y_{i}\right)$ and $P_{i+1}\left(x_{i+1},y_{i+1}\right)$, and $SS_{j}$’s two endpoints are $SP_{j}\left(x_{j},y_{j}\right)$ and $SP_{j+1}\left(x_{j+1},y_{j+1}\right)$, respectively. The segment-segment distance is $dist_{s}\left(P_{i},SP_{j}\right)$ can be calculated as follows.

The point-point distance includes the spatial distance and the temporal distance. The spatial-temporal distance $dist_{st}\left(P_{i},SP_{j}\right)$ between $P_{i}$ and $SP_{j}$ is calculated as shown in Equation (8):

(8) $$dist_{st}\left(P_{i},SP_{j}\right)=dist_{p}\left(P_{i},SP_{j}\right)+t\times dist_{t}\left(P_{i},SP_{j}\right)$$

where *t* is the time sensitivity parameter. The larger parameter *t* is, the more sensitive the distance to the time dimension is. When parameter *t* = *0*, the time dimension cannot be neglected.

**Figure 7:**
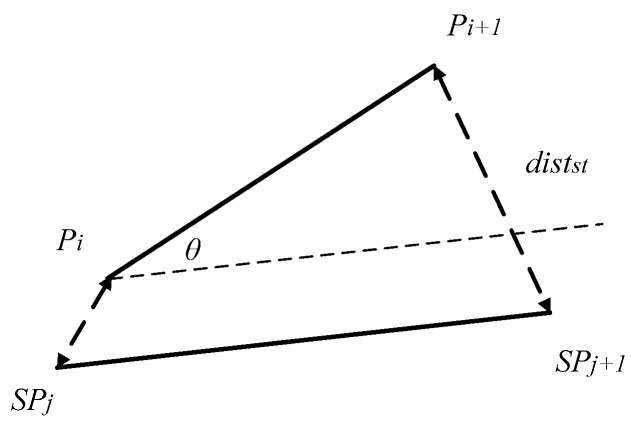
Segment-Segment Distance.

The segment-segment spatial-temporal distance is the sum of spatial-temporal distances between the two ends of the segments. $dist_{st}\left(S_{i},SS_{j}\right)$ represents the segment-segment spatial-temporal distance of $S_{i}$ and $SS_{j}$, as shown in Figure 7. $dist_{st}\left(S_{i},SS_{j}\right)$ can be calculated as follows:

(9) $$dist_{st}\left(S_{i},SS_{j}\right)=dist_{st}\left(P_{i},SP_{j}\right)+dist_{st}\left(P_{i+1},SP_{j+1}\right)$$

Then, $dist_{st}\left(S_{i},SS_{j}\right)$ and the angle distance can be combined to calculate the segment-segment distance. It computes the included angle between $S_{i}$ and $SS_{j}$ in Equation (10), denoted as θ:

(10) $$\theta =\left|\arctan2\left(y_{i+1}-y_{i},x_{i+1}-x_{i}\right)-\arctan2\left(y_{j+1}-y_{j},x_{j+1}-x_{j}\right)\right|$$

Under the same condition, if the included angle θ increases, $dist_{st}\left(S_{i},SS_{j}\right)$ should be multiplied with a certain time for the computation. Thus, θ should be integrated with $dist_{st}\left(S_{i},SS_{j}\right)$:

(11) $$dist_{s}\left(S_{i},SS_{j}\right)=f(\theta )dist_{st}\left(S_{i},SS_{j}\right)$$

where f(θ) can be computed in Equation (12):

(12) $$f(\theta )=\frac{dist_{smid}(S_{i},SS_{j})}{dist_{\max}(R,S)}\times (\omega +\theta )$$

where ω is an adjustable parameter and the shape negative factor. The greater ω, the less sensitive the distance to the shape factor. If there are no special requirements, let ω=1. $dist_{smid}(S_{i},SS_{j})$ is the spatial-temporal distance between midpoints $S_{i}$ and $SS_{j}$. $dist_{\max}(R,S)$ is the maximum temporal distance between any two points of trajectory sequences *R* and *S*. Furthermore, it makes no sense to compare the shapes of two trajectory sequences with a long distance. The shorter the distance, the more important the shape factor. Thus, $\frac{dist_{smid}(S_{i},SS_{j})}{dist_{\max}(R,S)}$ is used to dynamically adjust the weight of the shape factor.

### 4.4. SDTW Computation

After all of the segment-segment distances between trajectory sequences *R* and *S* have been calculated, the accumulative distance is computed derived from the idea of the DTW algorithm. Similar to the DTW algorithm, the similarity measurement of the SDTW is as follows:

(13) $$SDTW\left(R,S\right)=\{\begin{array}{c} 0\;\;\;\;\;,\;if\;n=0\;\;\;and\;\;m=0\\ \begin{array}{c} \infty \;\;\;\;\;,if\;n=0\;\;\;or\;\;m=0\\ dist_{s}\left(Head\left(R\right),Head\left(S\right)\right)+min\{\begin{array}{c} SDTW\left(T,Rest\left(S\right)\right)\\ SDTW\left(S,Rest\left(T\right)\right)\\ SDTW\left(Rest\left(T\right),Rest\left(S\right)\right) \end{array}otherwise \end{array} \end{array}$$

where *n* is the number of line segments on the trajectory sequence *R*, *m* is the number of line segments on the trajectory sequence *S*, and Head(R) indicates the first trajectory sequence $S_{1}$, and Rest(R) is the new trajectory sequence after *R* eliminated Head(R). That is to say, $dist_{s}\left(Head\left(R\right),Head\left(S\right)\right)$ represents the segment-segment distance between Head(R) and Head(S).

**Figure 8:**
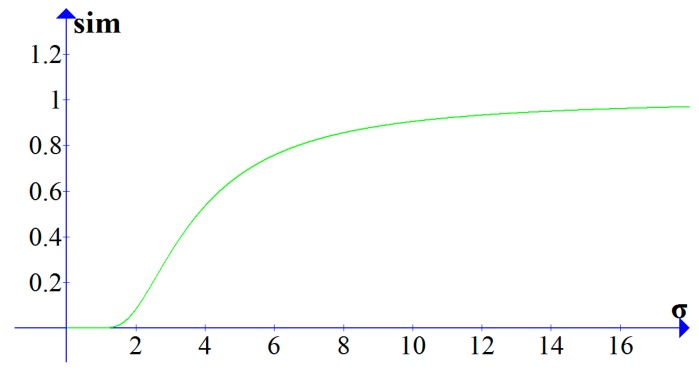
The sensitivity analysis about the value of σ.

The computed accumulative distance is negative correlation with the similarity between the trajectory sequences. The accumulative distances of different two trajectories will be quite different, thus, it cannot directly compare the accumulative distances. It is necessary to convert the accumulative distance into the range [0, 1], where 0 means the two trajectories are irrelevant and 1 means the two trajectories are the same. The conversion function uses the Gaussian kernel function. The conversion function is shown as Equation (14):

(14) $$Sim\left(R,S\right)=e^{-D^{2}(R,S)/2\sigma^{2}}\in [0,1]$$

where *D* represents the accumulative distance of *R* and *S*, σ is used to describe the sensitivity of the similarity to the accumulative distance. With the same *D*, the similarity is higher when σ is larger, and the similarity is lower when σ is small. In Figure 8, when d=10, with the increase of σ, the value of *sim* grows slowly within the range σ from 0 to 1.5. When σ is in the range from 1.5 to 6, the value of *sim* grows rapidly. When σ is greater than 6, *sim* grows slowly and approaches 1.

To sum up, the pseudocode of the SDTW algorithm proposed in the paper for the similarity computation for the two trajectory sequences is as follows:

As described in Algorithm 1, it first calculates the point-segment distance between each track point in the two trajectories according to Equation (3). If there is a temporal attribute in the trajectory data, it also needs to use Equation (7) to calculate the prediction distance. The segment-segment distance between each segment is then calculated using Equations (11) and (12). The subsequent calculation is the same as the DTW, and the final result is calculated using Equations (13) and (14) after initializing the accumulation distance matrix.

**Algorithm 1.** SDTW**Input:** Two trajectory sequences $R=\{P_{1},P_{2},...,P_{n}\}$ and $S=\{SP_{1},SP_{2},...,SP_{m}\}$ **Output:**
*Sim*, the similarity between *R* and *S* 1: ***for*** i = 0 ***to*** n           //Calculate all point-segment distance 2: ***for*** j = 0 ***to*** m 3: psDist[i][j] = caclPSDistance (p[i], sp[j]) 4: ***for*** i = 0 ***to*** n           //Calculate all prediction distance 5: ***for*** j = 0 ***to*** m 6: tDist[i][j] = caclTDistance (p[i], sp[j]) 7: ***for*** i = 0 ***to*** n − 1        //Calculate all segment-segment distance 8: ***for*** j = 0 ***to*** m − 1 9: sDist[i][j] = caclSDistance (s[i], ss[j], psDist, tDist) 10: init(matrix)           //Initial accumulation matrix 11: ***for*** i = 1 ***to*** n − 1       //Calculate accumulation distance 12: ***for*** j = 1 ***to*** m − 1 13: matrix[i][j] = sDist[i][j]+min (matrix[I − 1][j − 1], matrix[I − 1][j], matrix[i][j − 1]) 14: ***return*** gaussianKernel (matrix[n − 2][m − 2])

SDTW needs to traverse every trajectory point of the two trajectories when calculating the point-segment distance, the prediction distance and the segment-segment distance. caclPSDistance( ) is used to calculate the point-segment distance of two points in the two different trajectory sequences based on Equation (3). caclTDistance is used to calculate the prediction distance of two points in the two different trajectory sequences based on Equation (7). caclSDistance is to calculate the segment-segment distance based on Equation (11). caclPSDistance and caclSDistance only involve the calculated points or segments, without considering the other points or segments. The computational complexity of function caclPSDistance and caclSDistance is constant order O(mn). In Equation (7), the dichotomy is used to find the trajectory point interval where the predicted point is located. The computational complexity of caclTDistance is O(log(m+n)mn). The computational complexity of DTW is also O(mn). The computational complexity of SDTW is O(mn) for the trajectory data without the timestamp attribute, otherwise the computational complexity of SDTW is O(log(m+n)mn) for the data with the time-stamp attribute.

In this paper, the SDTW algorithm does not change the core concept of the DTW, and just replaces the DTW distance computation method with three types of distance. The SDTW can also use the lower limit of the DTW distance algorithm to improve the execution efficiency. Moreover, the point-segment distance, prediction distance and segment-segment distance can be integrated with the LCSS, EDR, and other algorithms to propose new approaches to the similarity measurement.

## 5. Performance Evaluation

### 5.1. Experimental Dataset and Metrics

The dataset used in the experiments are the GPS GeoLife Trajectories dataset from Microsoft Research [18] and CVRR Trajectory Analysis Dataset [19].

The experiments use the GeoLife dataset to compare DTW and SDTW. The dataset consists of GPS trajectory data of 182 users over five years, for a total length of 1,292,951 km, but the single trajectory sequence is too long, leading to rare trajectories with a high similarity, so the trajectory sequences in the dataset is split into about 500,000 shorter ones indexed with an R* tree. The dataset does not give the trajectory sequence relationship, so the experiment results can be evaluated through visual analysis.

**Figure 9:**
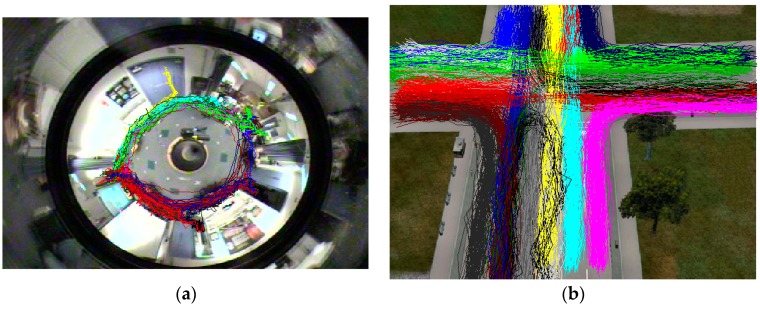
Labomni dataset (**a**) and Cross dataset (**b**).

The experiment uses the CVRR dataset to quantitatively analyze the accuracy, the robustness of the measurement algorithms, and the effects of the parameters. The dataset is specifically for assessing the trajectory analysis algorithm, and it mainly includes three types of trajectory data: the I5 dataset, the driving trajectory of a car on a two-way highway; the Labomni dataset, the data of people walking in the laboratory (Figure 9a); and the Cross dataset, the simulation of vehicles driving straight and turning at crossroads (Figure 9b). All of these datasets mark the clusters of each trajectory. These datasets can be clustered based on the trajectory similarity measure algorithm. The obtained clustering results can be compared with the correct clusters, which have been marked in the dataset, and give the accuracy analysis of the proposed SDTW algorithm. It should be noted that the I5 dataset is comprised of mainly linear trajectories, and most algorithms can obtain good results. Therefore, the experiments only use the Cross dataset and Labomni dataset.

In the experiments, the error rate is included as one of metrics to evaluate the performance.

**Definition 7** (**error rate**). *The error rate (ER) is the rate of wrongly-clustered trajectory sequences, which is different from CCR [20]. The lower the value, the higher the accuracy of the algorithm. Suppose the number of the known trajectory sequences is N, the total number of clusters is k, and the correct number of the sequence belonging to the class c is* $p_{c}$. *The error rate is defined as follows:*

(15) $$ER=1-\frac{1}{N}\sum_{c=1}^{k}p_{c}$$

### 5.2. Search Similar Trajectory

**Figure 10:**
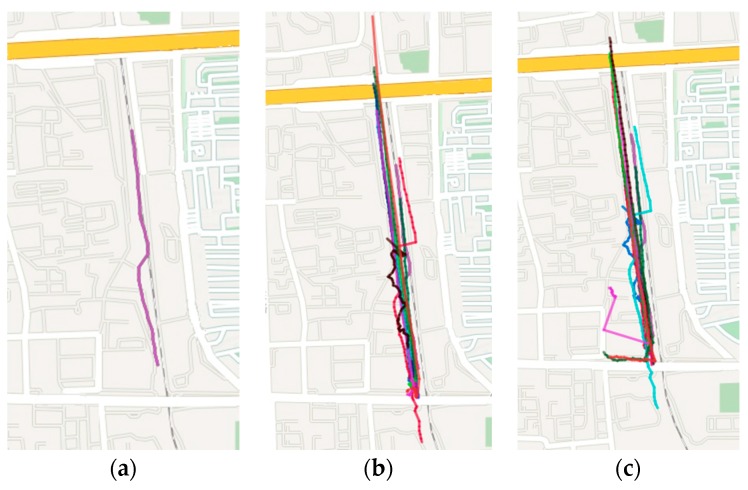
Query results of trajectory sequences with the SDTW and DTW algorithms. (**a**) The original query trajectory; (**b**) Query results of trajectory sequences with the SDTW; (**c**) Query results of trajectory sequences with the DTW algorithms.

The experiments use the same dataset of trajectory sequences for the trajectory queries. It can compute and obtain the top 15 most similar trajectory sequences with the original query trajectory in the dataset, through executing the SDTW and DTW algorithm, respectively. The computational results are visually displayed on the map. The original query trajectory is shown in Figure 10a, and the query results of the SDTW algorithm and DTW algorithm are shown in Figure 10b,c, respectively.

In Figure 10b, most of the query results of the trajectory sequence are close to the original query trajectory, and have high similarity in shape. In Figure 10c, many query results have low similarity in shape, compared with the original query trajectory. The reason is that the SDTW algorithm considers the shape factor of the natural trajectory and uses the point-segment distance to reduce the loss of the sampling method on the accuracy, so the SDTW algorithm is more accurate than the DTW algorithm.

### 5.3. Clustering Error Rate Comparison

The experiment is based on the CVRR dataset with *cluto* [21] as a clustering tool. *cluto* is a low-dimensional clustering and high-dimensional data software package for the analysis of the characteristics of various categories. *cluto* can provide a variety of optimized clustering algorithms, and support the trajectory clustering based on the similarity matrix.

**Figure 11:**
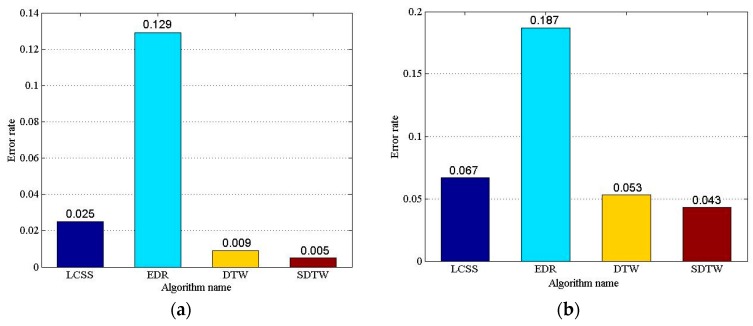
Comparison of Clustering Error rates. (**a**) Clustering error rates based on the Cross dataset; and (**b**) clustering error rates based on the Labomni dataset.

In order to evaluate the accuracy of similarity measurement, four algorithms, LCSS, EDR, DTW, and SDTW are selected to cluster the trajectory sequences. First, the trajectory similarity matrix of two datasets are generated with the similarity measurement algorithm. Then, it is clustered with agglomerative hierarchical clustering (AHC) and *rbr* with global optimization. Finally, the maximum ER of each dataset is regarded as the final result of clustering error rate. As to the LCSS and EDR algorithms, it is necessary to specify a threshold ε. In the experiments, LCSS and EDR algorithms should calculate the maximum ER with the threshold ε varying in range from 1 to 5, when the step is set to 1.0. As to the SDTW algorithm, it is necessary to specify the parameter ω. The SDTW algorithm should calculate the maximum ER with parameter ω varying from 1 to 10, when the step set to 1.0. It should be noted that letting the parameter σ in Gaussian kernel function $\frac{1}{N^{2}}\sum_{i}\sum_{j}sim_{ij}=0.1$ can produce very good clustering results [22]. Figure 11 illustrates the compared results of the clustering error rate with the four algorithms.

As shown in Figure 11, the algorithms with various datasets lead to various error rates, but the order of the error rate is the same. The LCSS, DTW, and SDTW can obtain good clustering results, and the EDR’s clustering effect is poor. The error rate of the SDTW is the lowest, and the error rate of LCSS is higher than that of the DTW. The error rate of the SDTW is 80%, 96.12%, and 44% lower than the LCSS, EDR, and DTW with the Cross dataset, respectively; and 35.82%, 77.01%, and 18.87% lower with the Labomni dataset, respectively. To sum up, the SDTW algorithm can obtain better accuracy than that of the DTW, LCSS, and EDR. The reason is that the SDTW algorithm introduces the prediction distance to convert the temporal distance into the spatial distance, considering the temporal distance factor. Additionally, SDTW introduces the segment-segment distance to improve the computation accuracy by adjusting the parameters of shape factors.

### 5.4. Noise Effect Analysis

To evaluate the robustness of various algorithm, noisy data at different levels is superimposed into the original trajectory sequence data. The noise rate reflects the deviation points ratio in the trajectory sequence. When the noise rate is λ (0 ≤ λ ≤ 1), it indicates that the trajectory points of 100λ% in the original data have been deviated to a certain extent. It is noted that due to noise randomness, the experiments are repeated 20 times and the average value is taken to ensure the accuracy of the results.

In the experiment, the variation of λ is in the range [0.1, 1] with the step 0.1. The deviation degree uses a random number. The deviation degrees of most deviation points are greater than the maximum threshold ε in the LCSS and EDR. The other parameters are set to the same value, as in the Section 5.3.

**Figure 12:**
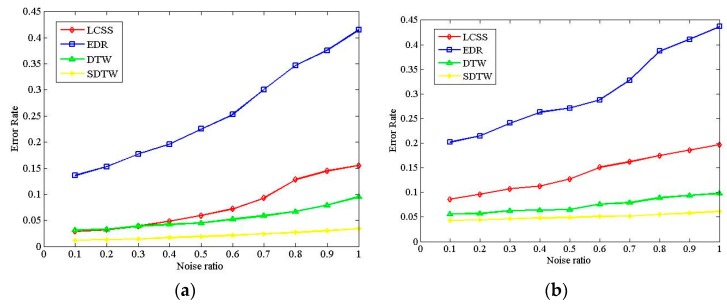
Comparison of noise effect on the algorithms. (**a**) Clustering error rates based on the Cross dataset; and (**b**) clustering error rates based on the Labomni dataset.

Figure 12a,b illustrates the experiment results with the Cross and Labomni datasets, respectively. The results of the clustering error rate are roughly consistent with the results in Section 5.3. It can be seen that the LCSS, DTW, and SDTW exhibit better accuracy than EDR, even in the case of noisy data. On the other hand, with the increase of the amount of noisy data, the clustering error rates of all algorithms increase gradually. From Figure 12, the maximum error rate of the LCSS, DTW, and SDTW is below 0.15 when the noise ratio varies from 0.1 to 1.0, which indicates good robustness to the noisy data for the above three algorithms. However, EDR exhibits poor performance on the robustness during the increase of noisy data.

On the other hand, In the Cross dataset, the average change ratio of ER in the LCSS, EDR, DTW, and SDTW is 23.35%, 14.88%, 14.15%, and 13.64%, respectively. In the Labomni dataset, that is 11.1%, 9.38%, 6.71%, and 4.21%, respectively. EDR and LCSS present poorer robustness than the DTW and SDTW. The conclusion that the robustness of the LCSS and EDR algorithms is better than the DTW when the deviation degree is greater than ε from [4] is not correct. The above conclusion is similar with [23]. The SDTW algorithm exhibits the best performance in terms of robustness, which benefits from the point-segment distance, which decreases the effect of sampling methods on the accuracy and also improves the robustness to noise.

### 5.5. Parameter Effect Analysis

In the SDTW algorithm, ω is an important parameter and determines the weight of the shape factor in the similarity computation. The experiments evaluate the effect of parameter ω varying from 0.3 to 20, as listed in Table 1. The experiment dataset is based on Labomni dataset. Two metrics are used to compute the error rate, AHC and *rbr*, respectively.

**Figure 13:**
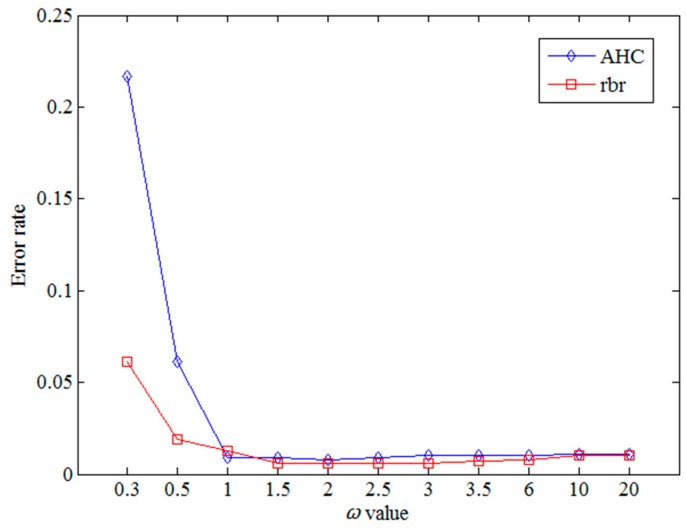
ER with various ω values.

From Table 1 and Figure 13, when the ω value is relatively small, the weight of the shape factor is quite large, which results in the high error rate. When the ω value lies in the range below 1.0, its small change will cause a great change in the error rate. When the ω value is larger than 1.0, its change will make little effect on the results. The results show no large difference with the optimal results. From the experimental results, the SDTW algorithm can obtain the optimal results with the appropriate value of parameter ω. Furthermore, the shape factors should be properly optimized, otherwise, the improper weights of the shape factors may result in poor performance on the error rate, as shown in Figure 13.

## 6. Conclusions

With the rapid development of sensor technology and the popularization of personal smart devices, GPS sensors are widely used to track moving objects. A trajectory similarity measure is one of the most important steps in trajectory data mining of human activity and vehicle moving patterns. Unfortunately, the main similarity measure algorithms with the trajectory data have been found to be inaccurate, highly sensitive to sampling methods, and have low robustness to the noise data. In order to solve the above problem, a segment-based dynamic time warping algorithm (SDTW) is proposed to measure the trajectory similarity. First, the proposed SDTW adopts the point-segment distance to reduce the sensitivity influence of the trajectory sampling method. Then, considering the temporal distance factor, SDTW introduces the prediction distance to convert the temporal distance into the spatial distance. Finally, SDTW introduces the segment-segment distance to improve the computation accuracy by adjusting the parameters of the shape factors. The experimental results indicate that the SDTW algorithm can obtain about 57%, 86%, and 31% better accuracy than the LCSS, EDR, and DTW, respectively. Meanwhile, the SDTW algorithm exhibits better robustness to the noise than that of the other algorithms.

## Figures and Tables

**Table 1:** Error rate with various ω values.

ω	**0.3**	**0.5**	**1**	**1.5**	**2**	**2.5**	**3**	**3.5**	**6**	**10**	**20**
AHC	0.061	0.019	0.013	0.006	0.006	0.006	0.006	0.007	0.008	0.010	0.010
*rbr*	0.217	0.061	0.009	0.009	0.008	0.009	0.01	0.01	0.01	0.011	0.011
